# Job-related factors associated with tobacco use among Chinese food delivery riders: A cross-sectional survey

**DOI:** 10.18332/tid/186930

**Published:** 2024-05-15

**Authors:** Chen Li, Dan Wu, Christopher Bullen, Jinsong Chen, Francis Cheung, Yonglin Zheng, Hongchen Luo

**Affiliations:** 1 School of Psychology, Shenzhen University, Shenzhen, China; 2 Shenzhen Humanities and Social Sciences Key Research Bases, Center for Mental Health, Shenzhen University, Shenzhen, China; 3 The National Institute for Health Innovation, School of Population Health, The University of Auckland, Auckland, New Zealand; 4 School of Law, Hangzhou City University, Hangzhou, China; 5 Department of Psychology, Lingnan University, Tuen Mun, Hong Kong

**Keywords:** smoking, job-related uncertainty stress, emotional exhaustion, food delivery rider, tobacco use

## Abstract

**INTRODUCTION:**

Food delivery drivers represent a rapidly growing occupational group in China in recent years. Their unique work patterns such as a complex work environment and high time-pressure may subject them to more severe tobacco use issues compared to other professions. This study aims to investigate the prevalence of tobacco use within this group and examine the underlying reasons behind it.

**METHODS:**

A cross-sectional, multistage sampling design was conducted to select 1879 food delivery riders from Guangzhou and Shenzhen. A self-administered questionnaire was used to collect the data from August to December 2022. Chi-squared analysis and binary logistic regression analysis, adjusted for factors including gender, education level, type of employment, alcohol use, job-related uncertainty stress, and emotional exhaustion, were used to explore the key factors associated with smoking among this occupational group.

**RESULTS:**

Altogether, 65.5% of individuals in this sample were smokers, with 69.5% among males and 26.2% among females. Factors found to be significantly associated with smoking behavior were male sex (AOR=5.48; 95% CI: 3.74–8.02), education level of junior high school or lower (AOR=1.60; 95% CI: 1.21–2.11), education level of senior high school (AOR=1.52; 95% CI: 1.18–1.95), full-time job (AOR=1.39; 95% CI: 1.18–1.80), alcohol use (AOR=3.91; 95% CI: 3.14–4.87), moderate level of job-related uncertainty stress (AOR=0.58; 95% CI: 0.42–0.81), high level of emotional exhaustion (AOR=1.57; 95% CI: 1.17–2.10) and moderate level of emotional exhaustion (AOR=1.52; 95% CI: 1.00–2.30).

**CONCLUSIONS:**

Demographic factors like gender, education level, job type, and substance use should be considered in designing smoking cessation campaigns for this group. Improving work conditions, reducing emotional exhaustion, and managing stress may also reduce smoking and enhance the well-being of these riders.

## INTRODUCTION

Cigarette smoking is a significant global health concern and a leading, preventable contributor to premature mortality^1^. In China, the world’s largest producer and consumer of tobacco products, approximately 25.8% of the entire population are smokers, with 48.8% of men and 2.3% of women among them^2^.

With the recent development of the gig economy^3^, workers in platform-enabled, peer-to-peer businesses have emerged as a rapidly expanding blue-collar occupational group. Work environment and job characteristics are important factors influencing smoking status^4^. Blue-collar workers and service workers in a range of countries have been shown to have relatively high smoking rates^5^. Chinese researchers have also delved into the correlation between occupations and smoking behavior. Yu et al.^6^ found that the smoking rate is highest among male operatives (61.4%), followed by males in commerce and service industries (53.7%). Prior research in this domain predominantly focused on conventional occupational categories, with relatively scant attention devoted to emerging job types, such as platform and gig economy workers. Among such employees, a significant subset comprises individuals involved in food delivery and ride-hailing services. Focusing on the food delivery sector, it is noteworthy that exclusively through the two primary platforms, Meituan and Ele.me, an excess of 7 million riders generated income in 2022^7^.

Job-related uncertainty may significantly stress food delivery riders. Stemming from a high volume of orders, diverse customer demands, and complex traffic conditions. These unique job characteristics could lead riders to use smoking as a coping mechanism for stress relief^8^. According to the Stress-coping Model^9^, stress is a key factor contributing to substance use, including smoking^10^. While previous studies have shown an increase in smoking behavior under stress^11^, there is limited research on the specific association between psychological uncertainty and smoking.

Another variable frequently explored in association with smoking is emotion^12^. The Affect Regulation Theory of Smoking posits that smoking influences emotional states^13^, particularly negative emotions. Previous research has primarily focused on negative emotions mediating the relationship between stress and smoking, indicating an increased risk of smoking^14^ associated with negative emotions. Limited research exists on the association between emotional exhaustion, a common workplace phenomenon, and smoking. However, it may also be an important factor influencing smoking behavior. Emotional exhaustion, characterized by chronic work-related strain^15^, may be influenced by factors such as emotional regulation, interpersonal dynamics, and individual traits, leading to job performance and subsequent turnover^16^. Previous studies have found a correlation between emotional exhaustion and smoking in other professions, possibly due to the regulatory effects of smoking on emotions and attention^17^.

Delivery drivers contend with a high volume of customers’ daily, long working hours and a fast-paced environment. Platform algorithms necessitate accepting as many orders as possible to maximize income and ensure customer satisfaction^18^. These conditions often lead to emotional exhaustion^19^. Consequently, we hypothesize that as emotional exhaustion levels increase, riders are more likely to smoke.

The aim of this study is to explore the smoking status among Chinese food delivery riders and its associated influencing factors, thereby providing insights for smoking cessation interventions.

## METHODS

### Research design and participants

This study employed a cross-sectional, multistage sampling design and employed a convenience-sampled questionnaire survey approach^20^.

In the first stage, two first-tier cities in the Guangdong Province, China, namely Shenzhen and Guangzhou, were chosen based on their high level of economic development, fast-paced lifestyle, and advanced food delivery industry.

In the second stage, 5 main districts in Shenzhen and Guangzhou were selected, and within each district, several streets were chosen to ensure geographical distribution.

In the third stage, data were collected through individual interviews using a structured questionnaire (Supplementary file) that typically lasted around 15 minutes, after obtaining consent from the participants. Survey administrators approached 1949 food delivery riders on the streets, distributed questionnaires, and offered opportunities for clarifying any questions while allowing ample time for completion. To enhance data reliability, the survey was completed anonymously, and participants were encouraged to provide honest responses. As a token of appreciation, each rider received a small gift valued at 10–20 RMB upon completing the questionnaire.

In the fourth stage, we excluded the top 5% of questionnaires that were completed in <250 seconds, resulting in a final count of 1879 valid questionnaires^21^.

The study protocol was approved by the Ethics Committee of Shenzhen University on 7 August 2022.

### Outcome variable

This study aimed to assess the current smoking status of food delivery riders. Therefore, the questions inquired about tobacco product use within the last 30 days. Respondents were categorized into two groups based on their current tobacco use^22^: 1) Non-smokers, referring to individuals who abstained from all smoked tobacco products; and 2) Current smokers, encompassing those who smoke tobacco products daily or on some days every week.

### Independent variables

*Demographic characteristics*

Age, gender, registered permanent residence, education level, and marital status were assessed.

*Job attributes*

Work platform, type of employment, social insurance, monthly income, working years, and daily working hours were measured.

*Alcohol use*

Tobacco and alcohol co-occurrence is highly prevalent^23^. This study measures alcohol consumption as a covariate. Self-reported alcohol use was based on the response to the following question: ‘Do you currently drink alcohol? (within one month)’. The response options included: 1) Yes, drink every day; 2) Yes, drink only on some days; and 3) No, do not drink. During data processing, items 1) and 2) were combined into ‘alcohol use-drinker’, and analyzed as a binary variable alongside ‘non-drinker’.

*Job-related uncertainty stress*

A self-developed scale consisting of 8 items was used to measure job-related uncertainty stress: This scale encompasses various domains, including uncertainty in the work environment, uncertainty in interpersonal relationships at work, uncertainty regarding personal occupational future development, and uncertainty related to organizational policies. Sample items in the scale include statements like: ‘Rapid advancements in intelligent technology pose a risk of unemployment due to industry transformations’ and ‘Feeling uncertain about my future career development’. All items were rated on a 5-point Likert-type scale, which ranged from 1 (no stress) to 5 (excessive stress). A higher total score indicated a higher level of job-related uncertainty stress. The scale demonstrated good reliability, with a Cronbach’s alpha coefficient of 0.94. Participants were categorized into low, moderate, and high groups. The high-stress group comprised individuals whose total scores were one standard deviation or more above the mean, while the low-stress group included those whose scores were one standard deviation or more below the mean. Participants with scores falling between the high and low groups were classified as the moderate group^24^.

*Emotional exhaustion*

Emotional exhaustion was measured by the subscale of Maslach Burnout Inventory (MBI)^25^. A Chinese version is available and research has demonstrated suitable psychometric properties, including validity and reliability of the scale^26^. A sample item includes: ‘I feel emotionally drained by my work’. The scale demonstrated strong reliability, with a Cronbach’s alpha coefficient of 0.94, indicating a high level of internal consistency. Similar to the aforementioned job-related uncertainty stress variables, a cutoff value of plus or minus one standard deviation was used^24^.

### Statistical analysis

All the analysis for this study was conducted with SPSS (version 26.0). Descriptive statistics were computed to ascertain the proportions of smokers and other relevant variables within each demographic category.

The reliability of the Emotional Exhaustion Scale and the self-designed Job-related Uncertainty Stress Scale were assessed using Cronbach’s alpha coefficient.

To examine the relationships between the independent variables (including demographic information, job attributes, job-related uncertainty stress, and emotional exhaustion data) and the outcome variable (smoking behaviors), chi-squared tests were used.

Following this, binary logistic regression analysis was conducted to identify the primary associations of the dependent variable; models were presented using odds ratios along with their corresponding 95% confidence intervals. Four regression models were constructed to explore the relationships between the respective variables and the dependent variable, as detailed in Tables 1 and 2. Model 1 included statistically significant sociodemographic factors (p<0.05), such as gender, education level, type of employment, and alcohol use. Models 2 and 3 added job-related uncertainty stress and emotional exhaustion, respectively. The comprehensive Model 4 combined all previous variables. Adjusted odds ratios (AORs) were used throughout to assess the impact on smoking behavior. Additionally, the Spearman rank correlation was employed to verify the association between job-related uncertainty stress and emotional exhaustion among smokers and non-smokers.

**Table 1:** Demographic characteristics of all participants by tobacco use status in a cross-sectional study in Guangzhou and Shenzhen, China, 2022 (N=1879)

*Characteristics*	*All n (%)*	*Smokers n (%)*	*χ²*	*p*
**Demographics**				
**Total**	1879 (100)	1231 (65.5)		
**Age** (years)			0.219	0.994
<20	165 (8.8)	110 (66.7)		
20–24	556 (29.6)	365 (65.6)		
25–29	521 (27.7)	341 (65.5)		
30–34	374 (19.9)	242 (64.7)		
≥35	263 (14.0)	173 (65.8)		
**Gender**			129.762	<0.001
Male	1707 (90.8)	1186 (69.5)		
Female	172 (9.2)	45 (26.2)		
**Registered permanent residence**			3.236	0.072
Rural	1457 (77.5)	970 (66.6)		
Urban	422 (22.5)	261 (61.8)		
**Education level**			30.391	<0.001
Junior high school and lower	538 (28.6)	385 (71.6)		
High school	758 (40.3)	515 (67.9)		
Junior college and university	583 (31.0)	331 (56.8)		
**Marital status**			0.219	0.896
Unmarried	1277 (68.0)	838 (65.6)		
Married	525 (27.9)	341 (65.0)		
Divorced and widowed	77 (4.1)	52 (67.5)		
**Job attributes**				
**Work platform**			4.410	0.110
Online platforms	550 (29.3)	341 (62.0)		
Agency companies	1035 (55.1)	696 (67.2)		
Self-employed	294 (15.6)	194 (66.0)		
**Employment status**			22.990	<0.001
Part-time	420 (22.4)	234 (55.7)		
Full-time	1459 (77.6)	997 (68.3)		
**Social insurance**			0.167	0.683
Paid	1384 (73.7)	903 (65.2)		
Unpaid	495 (26.3)	328 (66.3)		
**Monthly income** (RMB)			13.666	0.003
≤3999	274 (14.6)	156 (56.9)		
4000–7999	835 (44.5)	553 (66.2)		
8000–11999	622 (33.1)	413 (66.4)		
≥12000	148 (7.9)	109 (73.6)		
**Working years**			4.287	0.369
<1	1015 (54.0)	686 (67.6)		
1–2	357 (19.0)	226 (63.3)		
2–3	226 (12.0)	143 (63.3)		
3–4	126 (6.7)	78 (61.9)		
>4	155 (8.2)	98 (63.2)		
**Daily work hours**			12.521	0.006
<4	139 (7.4)	75 (54.0)		
4–8	176 (9.4)	107 (60.8)		
8–12	1226 (65.2)	815 (66.5)		
>12	338 (18.0)	234 (69.2)		
**Alcohol use**			174.524	<0.001
Drinker	884 (47.0)	715 (80.9)		
Non-drinker	995 (53.0)	516 (51.9)		
**Job-related uncertainty stress^a^**			9.783	0.008
Low	293 (15.6)	193 (65.9)		
Moderate	1313 (69.9)	837 (63.7)		
High	273 (14.5)	201 (73.6)		
**Emotional exhaustion^a^**			11.541	0.003
Low	339 (18.0)	201 (59.3)		
Moderate	1220 (64.9)	800 (65.6)		
High	320 (17.0)	230 (71.9)		

Statistical significance was determined using the χ² test to derive the p.

a The cutoff value is the standard deviation score. Total scores above one standard deviation indicate higher levels of job-related uncertainty stress and emotional exhaustion. RMB: 1000 Chinese Renminbi about US$140.

**Table 2:** Binary logistic regression results of factors associated with smoking among all participants in a cross-sectional study in Guangzhou and Shenzhen, China, 2022 (N=1879)

*Variables*	*AOR (95% CI) ^a^*			
	*Model 1^b^*	*Model 2^b^*	*Model 3^b^*	*Model 4^b^*
**Gender**				
Male	5.25 (3.59–7.67)***	5.36 (3.67–7.83)***	5.23 (3.58–7.65)***	5.48 (3.74–8.02)***
Female ®	1	1	1	1
**Education level**				
Junior high school and lower	1.59 (1.21–2.09)***	1.57 (1.19–2.07)***	1.60 (1.21–2.10)***	1.60 (1.21–2.11)***
Senior high school	1.49 (1.16–1.91)**	1.50 (1.17–1.93)***	1.49 (1.16–1.91)**	1.52 (1.18–1.95)***
Junior college and university ®	1	1	1	1
**Employment status**				
Full-time	1.35 (1.05–1.74)*	1.39 (1.08–1.79)*	1.36 (1.06–1.75)*	1.39 (1.08–1.80)*
Part-time ®	1	1	1	1
**Alcohol use**				
Drinker	3.87 (3.11–4.81)***	3.92 (3.15–4.88)***	3.80 (3.06–4.73)***	3.91 (3.14–4.87)***
Non-drinker ®	1	1	1	1
**Job-related uncertainty stress^c^**				
High ®		1		1
Moderate		0.61 (0.44–0.84)**		0.61 (0.42–0.89)**
Low		0.86 (0.58–1.29)		1.05 (0.66–1.67)
**Emotional exhaustion^c^**				
High			1.51 (1.06–2.16)*	1.57 (1.17–2.10)**
Moderate			1.29 (0.98–1.68)	1.52 (1.00–2.30)*
Low ®			1	1

a AOR: adjusted odds ratio.

b Statistically significant sociodemographic factors for smoking, including gender, education level, employment type, and alcohol use, were included in Model 1. Models 2 and 3 expanded on Model 1 by integrating job-related uncertainty stress and emotional exhaustion, respectively. Model 4, the full model, combined all covariates.

c The cutoff value is the standard deviation score. Total scores above one standard deviation indicate higher levels of job-related uncertainty stress and emotional exhaustion.

* p<0.05.

** p<0.01.

*** p<0.001.

® Reference categories.

All statistical tests were performed at a 95% confidence interval, with a significance level set at p<0.05, and all statistical tests conducted throughout the study adhered to a two-tailed criterion.

## RESULTS

### Sample characteristics

Table 1 provides an overview of the study sample’s characteristics (without weighting). In summary, the predominant demographic features of the sample include a high proportion of males (90.8%), unmarried participants (68%), with an education level of senior high or lower (68.9%), in full-time employment (77.6%), working an average of 8–12 hours per day (65.2%), and consuming alcohol (53%). The average age of the participants was 27.5 years, with an age range 18–56 years.

### Factors associated with current tobacco use

In this sample, almost two-thirds were smokers, with smoking significantly higher in men than women and in those with full-time work, lower education level and lower incomes. Almost three-quarters of the smokers reported experiencing high job-related uncertainty stress levels and 72% of individuals facing high emotional exhaustion were also smokers.

**Figure 1:**
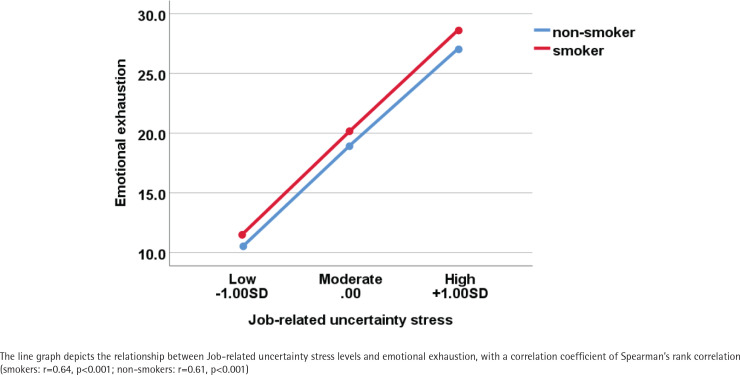
Correlation between stress, emotional exhaustion, and smoking in a cross-sectional study among all participants in Guangzhou and Shenzhen, China, 2022 (N=1879)

In univariate analysis, gender, education level, type of employment, alcohol use, job-related uncertainty stress, and emotional exhaustion were all found to be associated with smoking (Table 2). In the bivariate analyses, in Model 1, a significant gender difference in smoking behavior was observed, with males being more likely to smoke (AOR=5.25; 95% CI: 3.59–7.67) than females. Furthermore, when compared to smokers with a college education or higher, those with an education level of junior high or lower, or senior high, were more likely to smoke (AOR=1.59; 95% CI: 1.21–2.09 and AOR=1.49; 95% CI: 1.16–1.91, respectively). Additionally, individuals engaged in full-time work (AOR=1.35; 95% CI: 1.05–1.74) and those who consumed alcohol (AOR=3.87; 95% CI: 3.11–4.81) were also more likely to smoke. In Model 2 and Model 3, a moderate level of jobrelated uncertainty stress was found to be negatively associated with smoking (AOR=0.61; 95% CI: 0.44–0.84). While individuals experiencing high emotional exhaustion were more likely to smoke (AOR=1.51; 95% CI: 1.06–2.16). The adjusted full model (Model 4) indicated that individuals who perceived higher emotional exhaustion were more likely to smoke than those with lower scores (AOR=1.57; 95% CI: 1.17–2.10). Moderate levels of job-related uncertainty stress showed a negative correlation with smoking (AOR=0.61; 95% CI: 0.42–0.89). Figure 1 illustrates a positive correlation between job-related uncertainty stress and emotional exhaustion. Among smokers, the correlation coefficient between job-related uncertainty stress and emotional exhaustion was 0.64 (r=0.64, p<0.001), while among non-smokers, the correlation coefficient was 0.61 (r=0.61, p<0.001), indicating a moderate level of correlation in both groups.

## DISCUSSION

This study aligns with prior research findings, reinforcing the associations between smoking behavior and various demographic and occupational factors such as gender, blue-collar employment, lower education level, and concurrent substance use (in this case, alcohol consumption)^23^. A significant finding of this study is the high smoking prevalence among food delivery riders, encompassing both males and females, surpassing the national average^2^. Notably, the considerably higher smoking rate among the females (26.2%) is a striking contrast to the national adult female average of 2.3%^2^. Women are more susceptible to stress compared to men^27^, and in addition to similar work intensity as the male food delivery riders, females may also face additional pressures from childcare and household chores, which could lead them to use smoking to relieve stress. Furthermore, given the higher prevalence of smoking among the riders, increased exposure to smoking environments may also influence the females to smoke. These results warrant further investigation and emphasize the necessity for enhanced dissemination of smoking-related health knowledge and stricter workplace regulations within this population. The observed increased smoking rates among riders with lower education level suggest that promoting educational opportunities or continuing education within their occupational management could effectively mitigate smoking prevalence. Furthermore, the higher likelihood of smoking among full-time riders might be attributed to the extended daily work hours necessitated by full-time employment, emphasizing the importance of tailor-made tobacco control and education programs for this specific occupational group^28^.

Moreover, this study unveils a noteworthy positive correlation between heightened emotional exhaustion and smoking behavior. Alternatively, based on the Stress Coping Model^9^, this behavior could signify the establishment of a maladaptive coping mechanism – smoking – as a response to emotional exhaustion and work stress^29^ that progressively develops over time. Social factors may also contribute to smoking when exhausted, as riders engage in smoking and conversation with colleagues or friends as a form of social activity to alleviate fatigue.

The data also suggest that moderate job-related uncertainty stress levels may act as a protective factor against smoking. However, excessively high or low job-related uncertainty stress levels might indicate a greater propensity for smoking, although this relationship did not reach statistical significance in the present study. While the findings indicate a reduced likelihood of smoking occurrence in the moderate job-related uncertainty stress group compared to the high-stress group, this finding remains inconsistent with the hypothesis of our study. Therefore, further analysis of this variable should be conducted in future research to provide more clarity and insights.

An intriguing observation arises when considering the association between different job-related uncertainty stress groups and smoking: the high-stress group initially exhibits the highest smoking rate. However, after incorporating other variables into the model, the smoking rate in the high-stress group decreases, with the lowest likelihood of smoking observed in the moderate-stress group. This result contradicts previous research findings^13^. According to the Yerkes-Dodson law, a moderate level of stress leads to the highest levels of cognitive and task performance. Both excessively high and excessively low levels of stress can potentially decrease performance^30^. Perhaps riders experiencing moderate levels of stress are more focused on their work and maintain a more positive attitude, contributing to the observed lower smoking rates. Alternatively, job-related uncertainty stress, possibly distinct from other forms of stress, entails an element of uncertainty, which may have a positive aspect. Research suggests that individuals do not always desire a reduction in uncertainty^31^, which might reduce the tendency for delivery riders to alleviate stress through smoking behavior.

### Implications

For the issue of high smoking rates among food delivery riders, we propose the following policy recommendations. Firstly, there should be comprehensive monitoring of smoking prevalence within this group, while focusing on male food delivery riders is essential, the high smoking rate among the females should not be overlooked. Additionally, attention needs to be directed towards the smoking status of riders with lower education levels and those who work full-time. We suggest implementing targeted government and community initiatives to enhance smoking education and awareness among this demographic. Furthermore, we recommend actively engaging smokers in smoking cessation intervention programs.

Secondly, we encourage the establishment of smoke-free work environments and the elimination of smoking subcultures. Government departments should strengthen public place smoking regulations, urge companies to enact relevant policies, incorporating smoking bans into occupational health management regulations or standards, intensify restrictions on smoking among delivery personnel, and impose stricter penalties on non-compliant entities^32^. Meanwhile, attention should be given to the issue of secondhand smoke exposure. Smoking behavior among riders during food delivery not only fosters a smoking-friendly atmosphere, hindering the creation of smoke-free environments, but also exposes non-smoking pedestrians, colleagues, and other people whom the rider may come into contact with, to secondhand smoke^33^.

Finally, considering the correlation between stress, emotions, and smoking, we recommend providing psychological stress and emotion management training for this group. Designing and implementing psychological well-being initiatives will equip them with skills to cope with stress and negative emotions encountered in their work^34^.

### Limitations

Firstly, this study employed a convenience sampling method, focusing solely on riders from two first-tier Chinese cities. This approach may raise concerns about the generalizability and representativeness of the findings. Secondly, the distribution of questionnaires occurred during a unique period influenced by the COVID-19 pandemic, potentially impacting the psychological states and behavioral patterns of the participants. In particular, the implementation of contactless delivery policies presents a two-fold challenge for food delivery workers: balancing productivity with strict adherence to preventive measures during deliveries. The severity of the COVID-19 pandemic notably diminished delivery performance and capacity utilization^35^. This situation may contribute to heightened perceived pressure among food delivery workers, potentially resulting in an elevated frequency of smoking. Thirdly, the recruitment of participants involved street intercepts, wherein respondents had face-to-face interactions with researchers, potentially introducing social desirability bias in their responses. Lastly, while the analyses controlled for various potential confounding factors, there are other crucial variables such as cultural elements were not included in the scope of this research.

It is important to note that this was a cross-sectional study, therefore the findings of associations between a range of variables and smoking does not indicate a causal relationship; indeed, smoking may induce feelings of stress, especially when people crave nicotine^36^.

## CONCLUSIONS

The unique working patterns of food delivery riders may impact their tobacco usage patterns. In this study, demographic variables associated with smoking, including gender, education level, type of employment, and the presence of other substance use behaviors, should be considered in the formulation of relevant smoking cessation campaigns and intervention policies for this substantial occupational group. Furthermore, efforts to help food delivery riders establish a more conducive working environment, improve their work patterns, reduce emotional fatigue, and maintain appropriate stress levels, can contribute to a reduction in smoking behavior. These measures may play a vital role in promoting the overall well-being of this population.

## Supplementary Material



## Data Availability

The data supporting this research are available from the authors on reasonable request.
