# Supplementary material for: Job-related factors associated with tobacco use among Chinese food delivery riders: A cross-sectional survey
Source: Tob Induc Dis. 2024 May 15;22:10.18332/tid/186930. doi: 10.18332/tid/186930 (PMC11095091; doi:10.18332/tid/186930)
Supplement: Supplementary file 1 [file TID-22-79-s1.pdf]

Questionnaire

Demographics

Your age：

Your gender：

①Male ②Female

Your registered permanent residence:

①Rural ②Urban

Your level of education:

①Junior high school and below ②High school ③Junior college & university

Your marital status:

①Unmarried ②Married ③Divorced & widowed

Job attributes

Your work platform:

①Online platforms ②Agency companies ③Self-employed

What type of employment do you have:

①Part-time ②Full-time

Do you have social insurance:

①Yes ②No

What is your monthly income(yuan):

①less than RMB 3999 ② RMB 4000-7999

③ RMB 8000-11999 ④more than RMB12000

How many years have you been working:

①less than 1 year ②1-2 years ③2-3 years ④3-4 years ⑤ more than 4 years

How many hours do you work per day:

①less than4 hours ②4-8 hours ③8-12 hours ④more than 12 hours

Substance use

Do you currently drink alcohol (within one month):

① Yes, drink every day ②Yes, drink only on some days ③No, do not drink

Do you currently smoke cigarettes (within one month):

① Yes, smoke every day ② Yes, smoke only on some days ③ No, do not smoke

Job-related Uncertainty stress

Here are different stressors that people often face in everyday life. For each situation, please specify your level of stress.

	No Stress felt	A little stressed	Somewhat stressed	Quite stressed	Extremely stressed

1.Working plans are frequently disrupted by uncontrollable events, exemplified by the impact of the COVID-19 pandemic.					
2.The salary system is unreasonable, and the work income is unstable.					
3.Customer needs are diverse,and feedback and evaluations from customers are unpredictable.					
4.Work colleagues change frequently, and there is a lack of stable communication with both peers and customers.					
5.There is no fixed workplace, and the work environment is complicated and unfamiliar					
6. The rapid development of intelligent technology poses the constant risk of unemployment					
7. Imperfect industry regulations make it challenging to safeguard personal rights.					
8. Feeling confused about the future career development.					

Emotional exhaustion

Please evaluate and judge your work-related experiences and reactions based on your actual feelings and experiences, using the descriptions below.

	Never	A few times a year or less	Once a month or less	A few times a month	Once a week	A few times a week	Daily
1. I feel emotionally drained by my work.							
2. I feel used up by the end of the day.							
3. I feel fatigued when I have to get up in the morning to face another day on the job.							
4. Working with people all day is really a strain for me.							
5. I feel ‘burned out’ from my work.							

©2024 Li C. et al.
